# A hybrid deep learning framework using convolutional and transformer models for robust plant disease classification

**DOI:** 10.1038/s41598-026-38209-z

**Published:** 2026-02-18

**Authors:** Mohammed Mohsin Jawed, Farhan Ahmed Tufail, Mohd Zunaid Ahmed, Adaline Suji R, Priyanka Nallusamy, Kiruba Thangam Raja

**Affiliations:** 1 School of Computer Science and Engineering , Vellore Institute of Technology , Vellore, 632014 India; 2 School of Computer Science Engineering and Information Systems, Vellore Institute of Technology, Vellore, 632014 India

**Keywords:** Plant disease diagnosis, Hybrid deep learning, Convolutional neural networks, Vision transformer, EfficientNet-B7, Precision agriculture, Computational biology and bioinformatics, Engineering, Mathematics and computing, Plant sciences

## Abstract

Plant diseases continue to pose a significant threat to worldwide food security, resulting in notable yield reductions and economic consequences. Automated disease diagnosis through machine learning has arisen as a potential solution; nevertheless, current methods frequently have difficulty in capturing both detailed local attributes and overarching contextual patterns found in plant leaf images. This study presents a thorough comparative examination of conventional and deep learning methods—such as Convolutional Neural Networks (CNNs), Vision Transformers (ViTs), YOLO, Support Vector Machines (SVMs), and Random Forests—for the classification of multi-class plant diseases. To overcome the constraints of individual CNN and transformer models, a new hybrid framework that integrates EfficientNet-B7 for strong spatial feature extraction with a Vision Transformer (ViT-B16) for comprehensive contextual modeling is suggested. The system is assessed on an extensive dataset consisting of 21,534 images covering 38 classes of plant diseases and healthy specimens. Experimental findings show that the suggested hybrid model reaches an accuracy of 98.13%, surpassing standalone CNN baselines and other rival models, while consistently achieving high precision, recall, and F1-scores for all classes. The results emphasize the success of combining convolutional and transformer-based models for scalable and precise plant disease detection, aiding the creation of smart decision-support systems for precision farming.

## Introduction

Agriculture is the foundation of food security in the world, but plant infections still cause massive losses of crops as much as 20–40 every year, hence posing a threat to economic stability and global food supply^1,2^. Plant diseases which are caused by a variety of pathogens like fungus, bacteria and viruses not only reduce the crop production but also compromise the quality of the produce thereby causing massive economic losses to the farmers and supply chain losses on international scale. The traditional methods of disease detection, based on the analysis and evaluation of diseases by humans and the use of a laboratory, require a lot of work and time, have subjectivity, and ultimately may lead to different diagnoses and delay the required actions^3,4^. In addition, these traditional methods require the use of professional plant pathologists, which are scarce in most areas, and in developing countries where agriculture is the major means of livelihood^5^.

The recent development of machine learning and deep learning methods has brought new possibilities in automation of plant disease detection. CNNs have also been particularly useful in image-based diagnostics where they automatically obtain layered feature representations of the unprocessed image^6^. The initial research, e.g., by Mohanty et al.^1^ and Sladojevic et al.^3^, demonstrates the effective application of CNN-based models to different categories of plant diseases. These systems obtain local features like edges, textures and patterns that are instrumental in the identification of disease symptoms. Nevertheless, their achievements are bound to their partiality in terms of local receptive fields. CNN architectures pooling layers although having the benefits of reducing the sizes of the feature maps and simplifying the computational burden, may lose spatial details that are crucial to identifying small or diffuse disease signals in large regions of a plant^7^. Differently, Vision Transformers (ViTs) are also a novel architecture that performs well in the global contextual capture information, through the use of self- attention. In contrast to CNNs, ViTs operate on images by dividing them into patches and predicting inter-dependences between these patches, keeping long-range spatially related images intact^8^. The challenge, however, has been the high computational demands as well as the fact that ViTs require very large datasets to perform competitively in practical agricultural settings. These limitations provide a system of balance between the local details of the fineness of details and the information of the whole contextual sense.

The proposed research is inspired by these observations in an attempt to bridge the gap between local and global feature extraction and propose a new hybrid architecture that makes use of the advantages of both CNNs and Vision Transformers (ViTs). We particularly use the EfficientNet-B7 as it has a powerful extraction of local features in an efficient manner, and the ViT B16 as the model that has the potential of capturing global dependencies through the self-attention mechanisms. The hybrid model is designed in such a way that it leverages the advantage of CNNs in identifying fine-grained patterns in addition to considering the contextual richness that ViTs offers. This is achieved by converting CNN-learned feature maps into an embedding that can be consumed by the transformer and by tokenizing and positional encoding the features to allow discovery of complex relations in features across the image^9^.

The main contributions of this work are summarized as follows:

(i) Exceptional Novel Hybrid Deep Learning Model with superior Accuracy:

A hybrid architecture that merges the powerful feature extraction capability of EfficientNet-B7 with the powerful global context modeling capability of the Vision Transformer (ViT-B16) has been developed. This model’s overall classification accuracy was an impressive 98.13% across a diverse dataset of 38 different classes of plant diseases, indicating that it is a robust and practical model.

(ii) Demonstrated Performance Superiority over Stand-alone Architectures:

Comparative analysis proved superiority of the proposed hybrid model over traditional standalone CNN and pure transformer models. The combination of EfficientNet and ViT components led to tangible performance improvements, outperforming with an accuracy improvement of up to 1.2% for standalone counterparts.

(iii) Architecturally Efficient Design for Comprehensive Feature Capture:

The hybrid architecture is an efficient way to capture both the local fine-grained features (through CNNs) and the global contextual dependencies (through the transformers). This improved and comprehensive feature extraction capability was realized without a substantial increase in architectural complexity or computational overhead to ensure that the model is practical for deployment.

Our methodology starts with the careful collection and preprocessing of high- quality images of plant leaves, which are normalized to a resolution of 224 × 224 pixels and normalized to ensure consistent input data. To handle the variability that exists in natural images, various techniques of data augmentation are widely used, which include random rotations, flips, and zoomed which increases the robustness and generalization of the model^10^. After preprocessing, the images are passed through the CNN backbone (EfficientNet-B7), which extracts deep feature maps that contain localized information that is important for detecting disease-specific patterns. However, realizing that CNNs find it difficult to remember global context, a 1 × 1 convolution operation is applied to project these feature maps into a higher dimensional embedding space which is then tokenized to feed into the Vision Transformer encoder^11^.

During the transformer phase, the feature maps are transformed into a sequence of tokens, and a learnable classification token (CLS token) is inserted in the initial position of this sequence of tokens. To keep the spatial context that is lost while the feature maps are flattened, learnable positional embeddings are added to each token. The Vision Transformer encoder then takes this sequence through multiple self-attention layers, where the model can aggregate the information from the whole image and capture long-range dependencies^12^. The output of CLS token which is now equipped with holistic representation of the input image is passed through dense (fully connected) layer and then fed with softmax function to get the probability distribution of different classes of diseases.

We take advantage of transfer learning and fine-tune a pre-trained EfficientNet-B7 model on our highly-annotated dataset of plant disease images. Training was conducted using batch size 32, initial learning rate 0.003 and for 10 epochs. The dataset is divided into training (70%), validation (20%) and testing (10%) subsets to ensure fair evaluation. We use Stochastic Gradient Descent (SGD) as our optimizer. Model efficacy is measured using accuracy, precision, recall and F1-score and, to prevent overfitting and ensure good generalization performance on varied image samples, we include cross-validation^13^.

In contrast to the traditional methods using CNNs only or the heavy computational cost of ViTs, our hybrid framework achieves the best balance - it combines the CNN-based feature extraction ability and the global context modeling ability of ViTs to provide better accuracy, robustness, and adaptability in the changing conditions of the field. This is especially important in agricultural settings where the early and accurate diagnosis of plant diseases can greatly reduce crop losses and improve overall yield. The enhanced performance of the hybrid model as evident from the accuracy level achieved (98.13%) which is a meaningful improvement over conventional models which usually exhibit lower performance levels under similar conditions^14^.

Furthermore, the deployment of the model on Google Colab allows for quick prototyping and collaboration between researchers. Colab’s environment with GPUs speeds up the training process, which makes it possible to work with large datasets and complex model architectures without relying on specialized hardware. This approach also improves reproducibility and scalability, allowing the model to be readily incorporated into practical use for agriculture applications and expanded to support additional categories of disease in the future^15^.

This work is a proposal to construct a hybrid CNN-ViT model featuring convolutional feature extraction and transformer-based global context modeling. The proposed architecture enhances the limitations of only CNNs and ViTs and leads to better classification performance across several plant disease classes. This research introduces a novel, hybrid architecture that synergistically integrates the strengths of Convolutional Neural Networks (CNNs) and Vision Transformers (ViTs) for advanced plant disease diagnosis. Termed as the CNN-ViT framework, the architecture is carefully designed to subvert the inherent limitations that have been observed in models that use only CNNs or ViTs.

The first element of the hybrid model exploits CNNs due to their common prowess in features extraction through hierarchy. CNNs can extract local, translational invariant properties (like texture, colour and shape of particular lesions) of the input plant images very well. This convolutional stage guarantees a powerful low-level representation of the visual features that are essential in the disease detection.

The framework incorporates the transformative capabilities of the ViT component after the local feature extraction. The ViT module does global context modelling. With the extracted CNN features as a sequence of patches or tokens, the transformer mechanism uses self-attention to learn the spatial interactions and connection between various parts of the plant leaf or stem. This universal consciousness is essential in identifying the complicated patterns of diseases that represent in extensive regions or are involved with delicate interactions among the local symptoms.

The combined architecture is therefore a win-win situation where the convolutional features tend to be very high-resolution and spatial specific to capture more details and the relational modeling of the transformers is long-range. Such a combination results in a more discriminative and comprehensive set of features.

The hybrid CNN-ViT framework is shown to have better classification results in a rather large and diverse set of plant diseases and various categories. The effectiveness of the hybrid model is confirmed by a comparative analysis with the current standalone models (pure CNN architectures (such as ResNet or VGG), and general implementations of ViT). It was demonstrated that such metrics as accuracy, precision, recall, and F1-score significantly improve, which makes this work a significant contribution to the sphere of automated plant disease identification and opens a way to even more accurate and reliable solutions in the sphere of agricultural technologies.

Overall, the suggested work will cover essential issues in plant disease diagnosis by proposing a hybrid CNN-ViT model, which will merge the most advantageous features of the two models. Our model is thoroughly trained and validated on a large-scale dataset, and its higher accuracy and robustness are due to the fact that architectural planning and training optimization were of priority. This work can play an important role in the research area of precision agriculture and offer some viable advantages to detect diseases early on, proactively manage crops, and the overall food security of the world in large scale.

## Taxonomy of plant disease detection techniques

The literature on plant disease detection methods has been broadly grouped according to the paradigm of learning and feature representation strategy. These methods are systematically grouped into four broad groups, including traditional machine learning-based, conventional deep learning-based convolutional, lightweight and transfer learning-based, and hybrid deep learning models which are a mixture of convolutional and attention-based processes.

Conventional machine learning methods use hand-designed feature extraction process, e.g., color histogram, texture descriptors and shape features, then apply support vector machine (SVM), random forest and k-nearest neighbor classification methods. Although these approaches showed decent performance in controlled settings, their reliance on manual feature engineering and weak generalization make them less effective in real-world settings with fluctuating illumination, background noise and disease severity.

The further development of deep learning led to the appearance of the convolutional neural network (CNN)-based models as a new paradigm of plant disease recognition. AlexNet, VGG, ResNet, DenseNet, and Inception are architectures that automatically extract hierarchical spatial features of leaf images, and their performance is much higher in terms of classification accuracy. Nevertheless, these models are mainly local feature extractors and cannot extract long-range dependence and global contextual content in complicated disease patterns.

MobileNet, EfficientNet, and SqueezeNet which are lightweight and transfer learning based models have become widely used to overcome computational constraints and deployment issues. These models have smaller models but with competitive accuracy; hence, it is applicable to real-time and edge-based agricultural applications. Although they are efficient, they may have a lower representational capability with visually similar classes of diseases.

More recently, hybrid deep learning models have been suggested to address the drawbacks of single CNN models by incorporating attention patterns or transformer architectures. The objective of these models is to integrate the powerful local feature extraction properties of CNNs with the global contextual modeling properties of Vision Transformer (ViTs). These hybrid architectures have been demonstrated to perform better in multiplexing the complex disease attributes in a variety of crop species. In this paper, the proposed Hybrid CNN-ViT model can be listed under this category, and it relies on EfficientNet-B7, which is employed to extract spatial features and Vision Transformer encoder, which is useful to model global features, leading to an improved performance of the classification on 38 plant disease classes.

## Literature review

The modern plans of diagnosis of plant diseases have received a significant development during the last decade, but a number of gaps still exist and should be further investigated. Mohanty, Hughes, and Salathe^1^ in their groundbreaking research presented a feasibility study to apply deep learning, in this case, CNNs to the raw plant images and used them to identify disease, which produced promising results. Nevertheless, they were limited in the size of the dataset and variability of the images, which impacted the applicability of their model. The foundation was later extended by Ferantinos^2^, who analyzed many different models based on deep learning, thus demonstrating that the architecture and fine-tuning process are very important in improving the diagnostic accuracy. Similarly, Sladojevic et al.^3^ also used deep neural networks to classify leaf images with decent recognition rates, but their method did not scale well under more heterogeneous data sets.

A comparative study of different deep learning models on the identification of plant diseases by Too et al.^4^ revealed that even with the potential of transfer learning to address the issue of data scarcity, the performance of the model remained significantly different based on the network trained, even though the pre-trained network was used . Ramcharan et al.^5^ also made contributions to the category of cassava disease detection by using deep learning to identify diseases in their data, with good accuracy but the method was not able to be applied to other crops. Barbedo^6^ examined the impact of the size and diversity of the dataset on the performance of deep learning and discovered that a more diverse and large training set had a significant positive impact on model accuracy, but with higher computational cost.

Following these initial advances, Brahimi, Boukhalfa and Moussaoui^7^ demonstrated the potential of CNNs in detecting plant diseases by introducing strong architectures; the models were frequently computationally intensive and less efficient in real-time models. Kamilaris and Prenafeta-Boldu^8^ gave an in-depth review of deep learning in agriculture by pointing out that, despite major progresses, the current models have shortcomings that include imbalanced data and poor feature extraction as they are being used in real-life settings. This work was expanded by Picon et al.^9^ who created mobile based deep convolutional neural networks to classify crop diseases under uncontrolled field conditions, but the real-time applications were constrained by hardware.

Jin et al.^10^, and Li et al.^11^ were interested in taking advantage of mobile devices to detect plant diseases, and made progresses of making these technologies more accessible and easily deployable, but did not achieve the same results in detecting subtle disease symptoms in different lighting and environmental conditions. A comparison of different transfer learning methods was presented by Zhang and Wu^12^, which demonstrated that even without any fine-tuning of a pre-trained network, the diagnostic performance can be significantly improved, yet, certain models, in particular, VGGNet-based ones, do not cope with computational efficiency. Huang et al.^13^ presented the lightweight CNN architectures designed to be used in mobile application, which, although being fast and energy-efficient, occasional sacrificed accuracy with the larger and more complex networks.

A detailed survey of deep learning methods in detecting plant diseases was made by Chen et al.^14^, and the development history was traced through basic CNN models up to the modern hybrid models. Rahman et al.^15^ and Ngan et al.^16^ carried out the implementation of real-time detection in embedded systems, with good performance in terms of speed improvement but with poor accuracy in the presence of heterogeneous fields. As a reaction to these restrictions, hybrid methods have been developed. Purohit et al.^17^ suggested a hybrid CNN-RNN model that tried to add temporal data to enhance disease detection but the complexity of the model and the cost of training made it impracticable. Khan et al.^18^ also improved the accuracy of detection using superior data augmentation techniques, but their approach was relatively resource-intensive.

In the works by Sa et al.^19^ and Paliwal et al.^20^, CNN-based models were used to achieve robust classification, which had high accuracy in controlled experiments; both studies cited difficulties in situations where two or more diseases could appear in an individual picture. More recently, Anusha et al.^21^, and Zhao et al.^22^ consider the application of transfer learning on state-of-the-art architectures such as EfficientNet in plant disease detection, and both articles have reported significant accuracy and efficiency improvements, but have experienced challenges in generalizing to novel disease types. A YOLO-based CNN method was created by Li et al. and did not have a high performance in the detection of tiny disease symptoms, but it provided fast detection ability^23^. Yu et al.^24^ and Guo et al.^25^ explored the ensemble techniques to integrate the merits of two or more models leading to improved performance indicators; however, the techniques increased the complexity of computations.

In addition, Abbas et al.^26^ and Tang et al.^27^ tried hybrid CNN- SVM models which enhanced the robustness of classification by combining the statistical learning with deep feature-extraction. Frames of end-to-end deep learning described by Zhang et al.^28^ and Singh et al.^29^ simplified the diagnostic process and were highly accurate in different types of diseases, but had issues with high inter-class similarity. Liu et al.^30^ and Oquendo et al.^31^ worked on the construction of efficient and lightweight architectures to real time plant disease detection with good accuracy rates but faced a setback in terms of feature granularity and environmental noise. Chen et al.^32^ and Nguyen et al.^33^ pursued the automatic detection methods, with advanced convolutional network, to the frontiers of early diagnosis of the disease.

Recently, Singh^34^ and Zhao et al.^35^ proposed hybrid-based architectures, which combine CNN and transformer blocks, with significant accuracy and resilience improvement, but more computational-resource-intensive. These hybrid methods were further advanced by Sun et al.^36^ and Roy et al.^37^ who added multi-scale convolutional networks and early detection mechanisms to these methods and Wu et al.^38^ who added remote sensing data to expand the disease monitoring area of these methods. Baltrusaitis et al.^39^ and Hernandez et al.^40^ played their role in creating deep learning systems which in addition to detecting diseases accurately, they are also scaleable to various types of crops. Kumar et al.^41^and Chen et al^42^. gave clues on the foundation of better CNN architectures and to use generative adversarial architecture in the supplement of training information respectively, leading to the establishment of more robust disease detection systems.

Gao et al.^43^ and Elmasry et al.^44^ designed custom deep learning models to classify specific crop diseases besides Barzegar et al.^45^ utilizing UAV imagery to enhance the accuracy of detection. Together, the studies by Chen et al.^42^, Gao et al.^43^, Elmasry et al.^44^, and Barzegar et al.^45^ underline the importance of the fact that the models of the plant disease should be able to perform not only with high precision but also with reliable functioning in various real-world conditions.

The main limitations of the previous approaches are overcome with our hybrid design combining the ability to capture fine-grained local features on a per-image level with Vision Transformers and a broad contextual understanding on a large scale through the use of Convolutional Neural Networks. With a precision of 98.13, it proves the power of the synergistic effect between these complementary mechanisms to detect plant diseases. Comparative analyses have shown that the hybrid model has always performed better than the top-ranking architectures, such as EfficientNet-B7, ViT B16,YOLO, Support Vector Machine (SVM), and Random Forest in terms of accuracy and resilience. The study presents a new and performing design that takes the plant disease diagnostics research to the next level and offers a scalable design that can be applied in real-life agricultural scenarios.

## Prerequisites

The proposed hybrid CNN + ViT model for plant disease diagnosis pre supposes that input images are captured at high resolution and under optimal lighting conditions to clearly reveal the disease symptoms on plant leaves.

### Hardware requirements

The system can be run on any regular computing platform, but its performance is greatly enhanced when a Graphics Processing Unit (GPU) with CUDA-based functionality is used. As much as the model can run on a CPU, it will need at least 8GB of RAM to store the image data and model checkpoints, and 16GB or more to effectively store large datasets. There is a need to have enough storage that can hold high-resolution images and numerous execution of model check points. Moreover, there must exist the stable high-speed internet connection, which will be needed to download and update necessary libraries and software dependencies.

### Software requirements

The hybrid model is written in Python 3.x and is based on deep learning systems like TensorFlow or PyTorch to comprise and teach the CNN as well as ViT modules. NumPy and Pandas are the main libraries that should be considered necessary as data manipulation tools, and OpenCV or Pillow are required to carry out tasks related to image processing. Such tools as Albumentations are used to facilitate data augmentation, which helps to enhance the learning procedure, introducing various augmentations that resemble real-world changes and enhance the generalization and resilience of the model. Lightweight web framework like Flask is used to create a user interface that can be accessed, and Jupyter Notebook is used as the main environment to work with the software and visualize the outcomes of the experiments and models.It is essential to have regular dependency management with the help of pip or conda to make sure that all software components are up-to-date and may be adjusted to new datasets and model demands.

These requirements would form the technical basis needed to be able to train, deploy, and maintain the hybrid CNN + ViT model to perform well at diagnosing plant diseases, such that the system would work best in a variety of operating conditions.

## Proposed work

This part describes the overall approach of our hybrid CNN + ViT model of plant disease diagnosis. The suggested framework is a combination of CNNs that are capable of capturing detailed local information with Vision Transformers that are capable of capturing the larger contextual information. The paper will be divided into several stages, which include the data collection and preprocessing, model structure and transfer learning, training and validation, and deployment. The steps are described in detail to show the general workflow and substantiate design decisions that were made to increase the diagnostic accuracy and strength. In contrast to current CNN Transformer models, which are based on patch-based tokenization or late feature fusion, the proposed architecture projects high-level CNN feature maps directly into the transformer embedding space, allowing dense interaction between local and global representations at the token level with small computational cost. This innovative method allows the token level interaction between the local and global representations in large density, which makes the input data better understood and the computational cost of the method is low.

### Data acquisition and preprocessing

A robust deep learning model is based on an excellent dataset. In that regard, we propose that the input images are of high quality and accurately represent the distinctive aspects of plant leaf disease. In this direction, we make use of the publicly available datasets with thousands of annotated images. These pictures are carefully selected with a variety of disease expression in the various conditions of the environment. However, one of the assumptions is that the collection of images is done in standardized conditions, that is they are taken under the same conditions in terms of lighting, focus, and background to reduce variability to the least possible which can have a negative impact on model performance. After the acquisition of the dataset, the preprocessing stage starts. All the images are reduced to a standard size of 224 × 224 pixels, which not only equalizes the size of the input, but also makes the training process less computationally intensive. The normalization of pixels is done by means of a scaling of the pixel values in the range [0, 1]. This normalization is essential in stabilization of the learning process by avoiding problems with different pixel intensity scales. As a form of data enhancement, we use a wide range of augmentation techniques, such as random rotations, horizontal and vertical flips, zoom, and small scale motions. This augmentation makes the training set effective, and it mimics the real world variations, thus, enabling the model to generalize more to unseen images. The resulting stage eventually ends up with a massive and diverse dataset which will form the required basis to train the hybrid model.

**Fig. 1:**
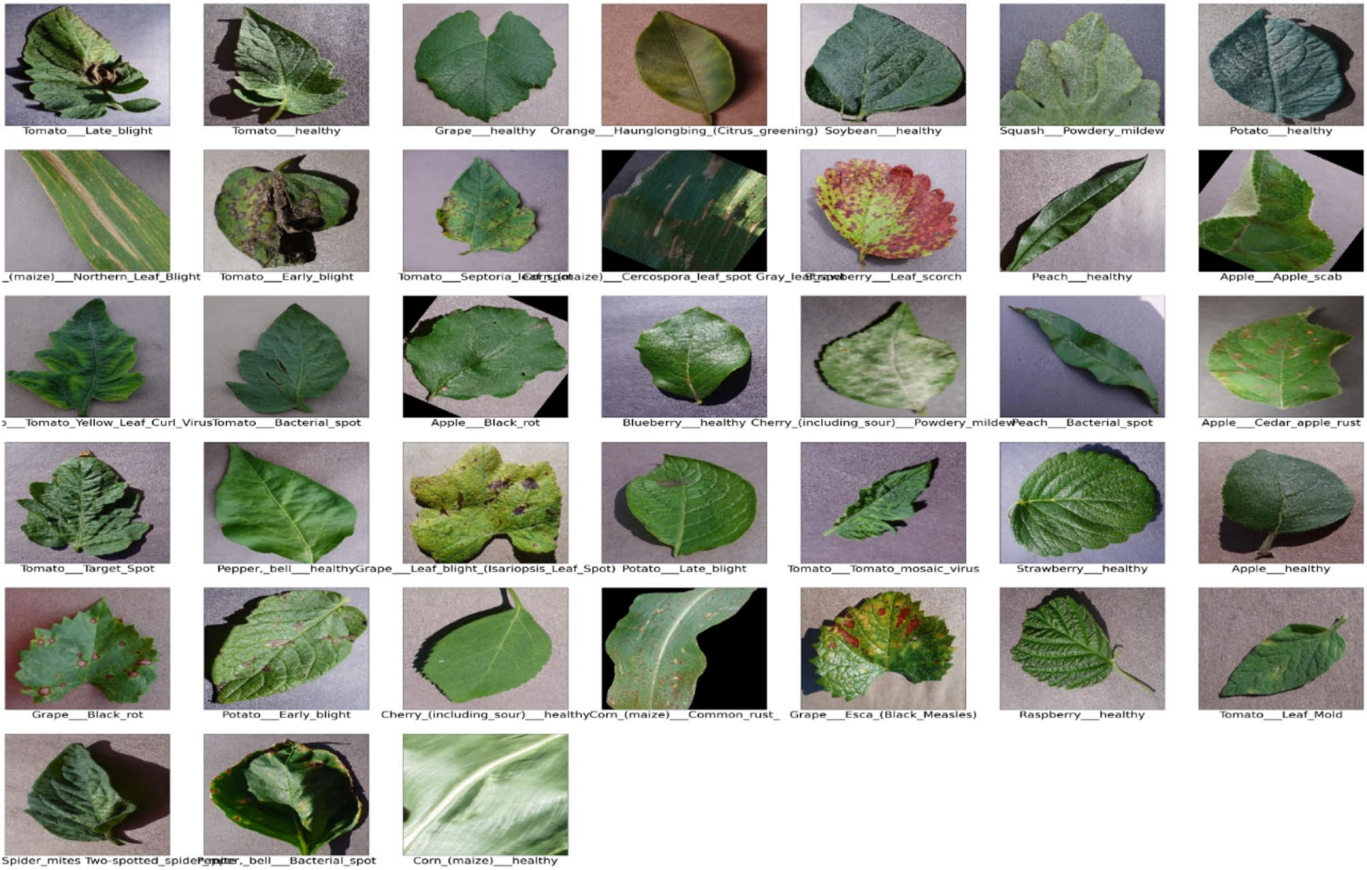
Sample images from the plant disease dataset

Representative sample images from the plant disease dataset illustrating dataset variability is shown in Fig. 1. The figure highlights variations in lighting conditions, background complexity, disease severity, and symptom appearance across different plant species and disease classes. Such variability motivates the use of a hybrid CNN–ViT architecture, where convolutional layers capture localized texture patterns while transformer-based self-attention models global contextual relationships.

### Model architecture and transfer learning

**Fig. 2:**
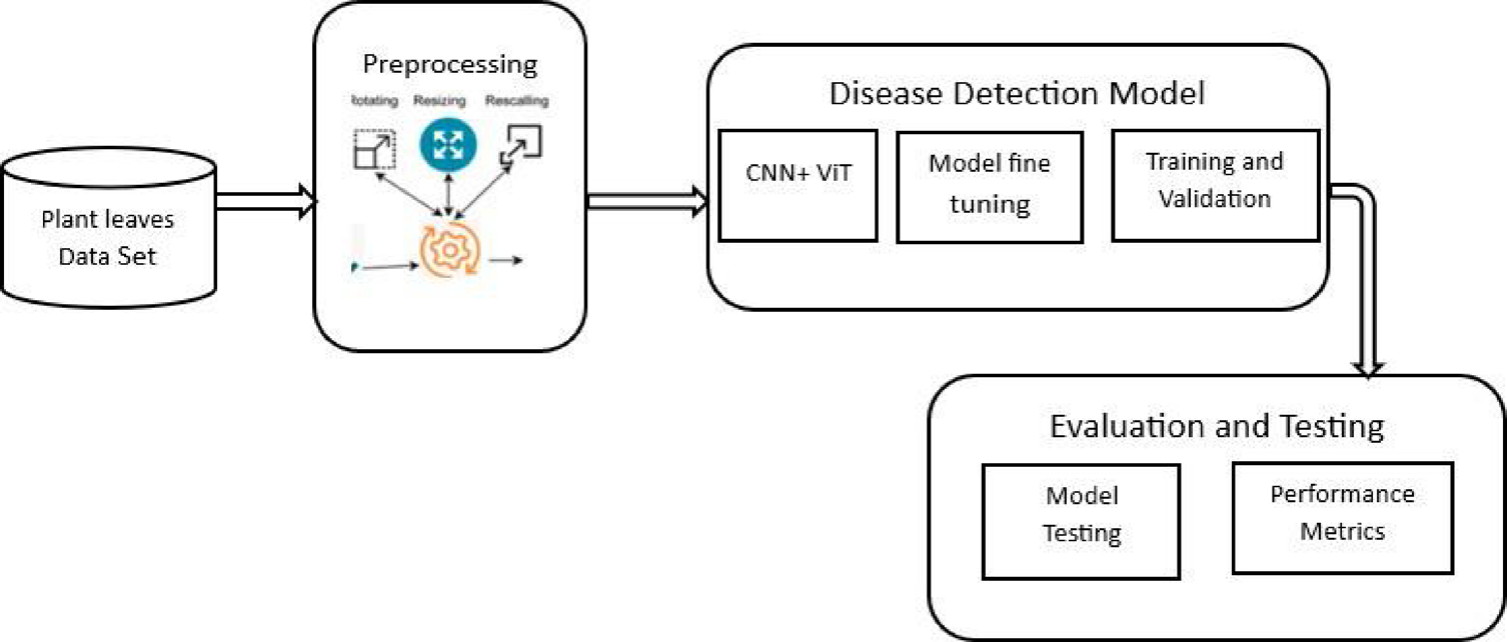
Process flow of proposed model.

The unique feature of our approach is its hybrid architecture through which the advantages of both Convolutional Neural Networks (CNNs) and Vision Transformers (ViTs) are merged in a synergistic way. Figure 2 represents the process flow of the proposed method.

During the first step, EfficientNet-B7, a CNN backbone that is pre-trained using ImageNet, is used as the architecture. Such pre-training allows extracting a wide-range of low-level features, such as edges, textures, and basic shapes. The CNN part has the role of producing rich and localized feature maps of the input images. Despite the high degree of success in detecting fine-grained patterns, CNNs are in general inefficient at detecting long-range dependencies within an image. To counter this, the architecture has included a Vision Transformer (ViT) that makes the model more capable of comprehending the global contextual relationships. After the CNN backbone has been fed on an image, it produces a high-dimensional feature map. To input these features to the ViT module, the CNN feature maps are projected to a vector space with a 1 × 1 convolution in the target embedding dimension of the ViT (e.g. 768 channels). This step of projection is important because it converts the CNN output in a form that can be processed by the transformer. After that, the spatial dimensions of the projected feature map are reduced to a linear sequence of tokens. A patch of the original image will be represented by each token that contains the localized features of the CNN.

Fig. 2 proposed hybrid CNN-ViT plant disease detection framework Workflow. The proposed hybrid CNN-ViT plant disease detector uses preprocessing tasks such as resizing, data normalization, and data augmentation on the plant leaf images. The preprocessed images are then forwarded to the hybrid CNN-ViT model, and EfficientNet-B7 results in high-level spatial features maps. These feature maps are then mapped into the transformer embedding space through a 1 × 1 convolution and rearranged into a series of tokens. It has a learnable classification (CLS) token before it and positional encodings thereafter to maintain spatial information. The vision transformer encoder works on the token sequence with multi-head self-attention in order to identify global contextual dependencies. The result that is produced of the CLS token is then fed into a fully connected layer to identify the disease. Standard performance metrics are used in order to perform model training, validation, and evaluation.

The initial token sequence is then occupied by the addition of a learnable classification token (CLS token). This token works to collect and encode data of the whole image in the course of the transformer during self-attention processes. Learnable positional embeddings are also used on each token to maintain the spatial structure of the original image, which transformers can not do. These embeddings are important in giving spatial awareness which is naturally present in CNNs and not present in transformer architectures. This message is last sent into the Vision Transformer (ViT) encoder to process it further and add the positional data.

ViT encoder is a series of sequential layers consisting of self-attention mechanisms combined with the feedforward neural networks. Through the process of self-attention, the encoder is able to identify and emphasize significant details in the entire image, effectively acquiring long-range relationships that are frequently overlooked by traditional CNNs. The product of this is a rich representation of features which combines the fine-grained local information as well as the global context. A feature vector of the CLS token is subsequently obtained, since it represents the overall diagnostic content of the image. This vector is passed onto a fully connected layer of classification, in which a softmax function is used to create probability scores of each of the disease classes.

The proposed system uses a CNN-ViT framework that aims at extracting local and global plant leaf features. EfficientNet-B7 is applied as CNN backbone to obtain hierarchical spatial feature like textures, edges, and patterns unique to the disease. A 1 × 1 convolution layer is used to project the extracted feature maps into an embedding space, which is compatible with transformers. Such embeddings are flattened into sequential tokens, augmented with positional encodings and a classification token, then run through a Vision Transformer encoder to learn long-range dependencies between the image. The last representation is transformed to a fully connected layer that has softmax activation in order to categorize the input into 38 disease and healthy categories.

#### Algorithm:

Hybrid CNN–ViT Disease Classification

1. Preprocess input leaf image (resize and normalize).
2. Extract spatial features using EfficientNet-B7.
3. Project features into transformer embedding space.
4. Tokenize features and apply positional encoding.
5. Model global dependencies using ViT encoder.
6. Classify disease using softmax output layer.

### Training and validation

After the architecture has been put in place, we then proceed with transfer learning to train the hybrid model. Our plant disease dataset is trained on the ImageNet-pretrained EfficientNet-B7 backbone such that the generic feature-extracting filters are specialized to plant pathology. Training is done using a data batch of 32, learning rate of 0.003, and a number of epochs is 30. The dataset is separated into 70% training, 20% validation and 10% final testing to allow strict evaluation and prevent overfitting. The model will also be applied using Stochastic Gradient Descent (SGD) as the optimization algorithm at the training stage because it is reliable and achieves optimal solutions, particularly when supplemented with other techniques such as momentum and learning rate scheduling. In order to compare the accuracy of predictions with the real class-labels, the categorical cross-entropy loss function is used. During training, different performance measures, such as accuracy, precision, recall and F1-score are computed, to monitor the performance of the model. Also, cross-validation is introduced with the aim to evaluate the generalization capability of the model in a strict manner, over the various partitions of the dataset. The training strategy that we employ involves data augmentation to increase the effective data set. These extensions enhance the stability of the network to a wide range of input fluctuations as well as learning of invariant features that are critical in accurate disease detection. Moreover, we make use of regularization methods - e.g., dropout and weight decay - to make the chance of overfitting even smaller. These methods together make up a model that is both precise and robust and can effectively work on a variety of unseen images.

### Software and implementation details

Data preprocessing, augmentation, model development, and training were performed using Python (version 3.10.6; https://www.python.org). The deep learning framework PyTorch (version 2.0.1; https://pytorch.org) was used for implementing and training the proposed hybrid CNN–ViT architecture. Image preprocessing and dataset handling were carried out using Torchvision (version 0.15.2; https://pytorch.org/vision).Pre-trained convolutional backbones were obtained using the timm (PyTorch Image Models) library (version 0.9.2; https://github.com/huggingface/pytorch-image-models), while the Vision Transformer (ViT) encoder was implemented using the Hugging Face Transformers library (version 4.31.0; https://huggingface.co/transformers).Model training and evaluation were accelerated using CUDA-enabled GPUs through Google Colaboratory (https://colab.research.google.com). Visualization and result analysis were conducted using Matplotlib (version 3.7.2; https://matplotlib.org) and NumPy (version 1.24.3; https://numpy.org). Tabular prediction outputs were generated using Pandas (version 2.0.3; https://pandas.pydata.org).

### Deployment on Google colab

To deploy it and continue experimentation, the whole model training and inference pipeline is run on Google Colab. Colab offers free access to GPUs that are vital in improving the speed of training deep learning models. Given the Colab usage also, sharing and collaboration is easily available, and other researchers can replicate our work and contribute to it. The model has been built into a working notebook with all the required code to preprocess the data, train the model, evaluate and predict the outcomes of the models.

In the Colab setting, users are able to post new images of plant leaves and instantly get diagnostic feedback, which is the suggested type of disease and the relevant confidence scores. The notebook can be interactive and can make real-time changes to hyperparameters and graphical representation of performance measures. Such interactive structure not only makes the process of evaluating the model easier but also offers an easy-to-use platform to make specific improvements and experiments.

### Summary of the proposed workflow

The proposed hybrid CNN + ViT model for plant disease diagnosis can be summarized as follows: The process starts by getting good quality plant leaf imageset, which are preprocessed (resizing, normalizing, data augmentation). Input images are processed through an EfficientNet-B7 CNN backbone in order to receive fine-grained local feature representations. A later projection step maps these features into an embedding space that can be used by the Vision Transformer after tokenization of the feature map and incorporating positional information. The tokenized input is passed to ViT encoder to learn global relations and the representation stored in CLS token is passed through a dense layer with softmax activation and is classified. The model is trained through transfer learning and using a curated dataset with performance optimization through strict training protocols and cross-validation. Finally, the system is implemented on Google Colab, providing an interactive and scalable platform for plant disease diagnosis.

## Result and discussion

Our CNN + ViT hybrid model was strictly tested on a large dataset of 21,534 high-quality images of plant leaves in 38 disease classes. Such pictures are based on the Kaggle new-plant-diseases-dataset repository which covers a wide variety of conditions and manifestations of a disease, including fungus, bacteria, and viral infections of different plants, including apple, tomato, grape, and corn. Before training the model, every image was processed through a common preprocessing pipeline, where each image was resized to 224 X 224 pixels, and put into the [0, 1] intensity range. Only the training set underwent several augmentation strategies, namely random rotations to ± 30, horizontal and vertical flips, and zoom to make it 0.8 or 1.2 times. These preprocessing actions assisted in simulating real-world variation and enhanced the model in its robustness to a great degree. A few of these measures that were used will be expressed in the In Eqs. (1–4), the True Positive (TP) represents a properly classified disease state with respect to a leaf image. When a leaf image is incorrectly labeled as a healthy leaf when it is in a disease category, it is called a False Negative (FN). A False Positive (FP) is an example of an incorrectly labeled image of a healthy leaf. Moreover, TN is an abbreviation that is translated as True Negative, which can be correct in identifying a healthy leaf in an image. The true positive rate (TPR) is the measure of the extent that the model can recognize a picture of a leaf as belonging to the correct disease category among all the positive pictures. Sensitivity is also known as TPR. Along with the generation of a considerable amount of FP, the high level of sensitivity means that the leaf images are quickly recognized as a disease representation. In order to calculate the accuracy, the total number of test photos divided by the TP plus TN is used to calculate the accuracy. It is observed that the F1 score is an easier method to measure the ability of the model to deal with class imbalances, especially in the cases where a large number of categories exist with different numbers of images.

1 $$Accuracy \: = \: \frac{TP \:+\:TN}{TP \:+\: TN \:+\: FP \:+\ FN}$$

2 $$Precision \: = \: \frac{TP}{TP \:+\: FP}$$

3 $$Recall \: = \: \frac{TP}{TP \:+\: FN}$$

4 $$Fl \: = \:2\: \star\: \frac{P \: \star \: R}{P \: +\: R}$$

The hybrid architecture suggested includes the advantages of two potent deep learning paradigms. The former, which is an EfficientNet-B7 model that is pre-trained on the Image dataset, is used as the backbone to extract local features. This network is skilled enough to obtain fine-grained visual features that include texture, color gradients, and edge orientations, which are essential in distinguishing the disease-specific symptoms on the leaves of plants. To ensure that the outputs of the next stage comply with the needs, the feature maps generated by EfficientNet -B7 are passed through a 1 × 1 convolutional layer that reshapes feature maps into a 768 dimensional embedding space. The resulting feature maps are reshaped into a sequence format where every token represents a certain spatial patch of the image.

**Fig. 3:**
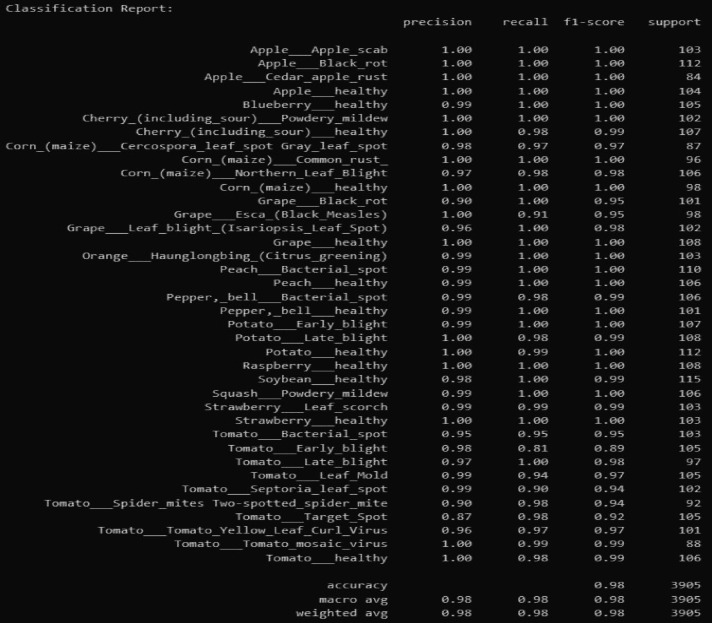
Classification Performance Metrics Report.

This sequence is predetermined with a learnable classification token (CLS) and positional embeddings to preserve the original spatial connections between patches. The augmented sequence of tokens is then passed through a multi-layer Vision Transformer (ViT B16) encoder, which incorporates self-attention mechanisms to combine information across the global context. The transformer can recover the long range dependencies by taking into account the local nature of the CNN, thus allowing the model to be able to comprehend the detailed features as well as the general structure of the diseased leaf. The last output of the CLS token is inputted into a fully-connected classification head, whose output is a softmax activation with a probabilistic prediction given on each of the 38 disease classes represented. The classification performance metrics report of the proposed model is presented in Fig. 3.

The model was trained using Google Colab using the support of GPU which was critical in controlling the high computational requirements of hybrid CNN-ViT architecture. The hybrid model was trained with the use of a stochastic gradient descent with momentum, the learning rate was set to 0.003, the batch size was 32, and the training was performed in 10 epochs. The data was divided as 70% train, 20% validate and 10% test. Both the training loss curve and validation loss curves were also tracked during the training and were shown to decrease steadily without significant over-fitting. Following the training, the model achieved a test accuracy of 98.13% and the detailed evaluation measures indicated high precision, recall, and F1 score in all disease categories.

**Fig. 4:**
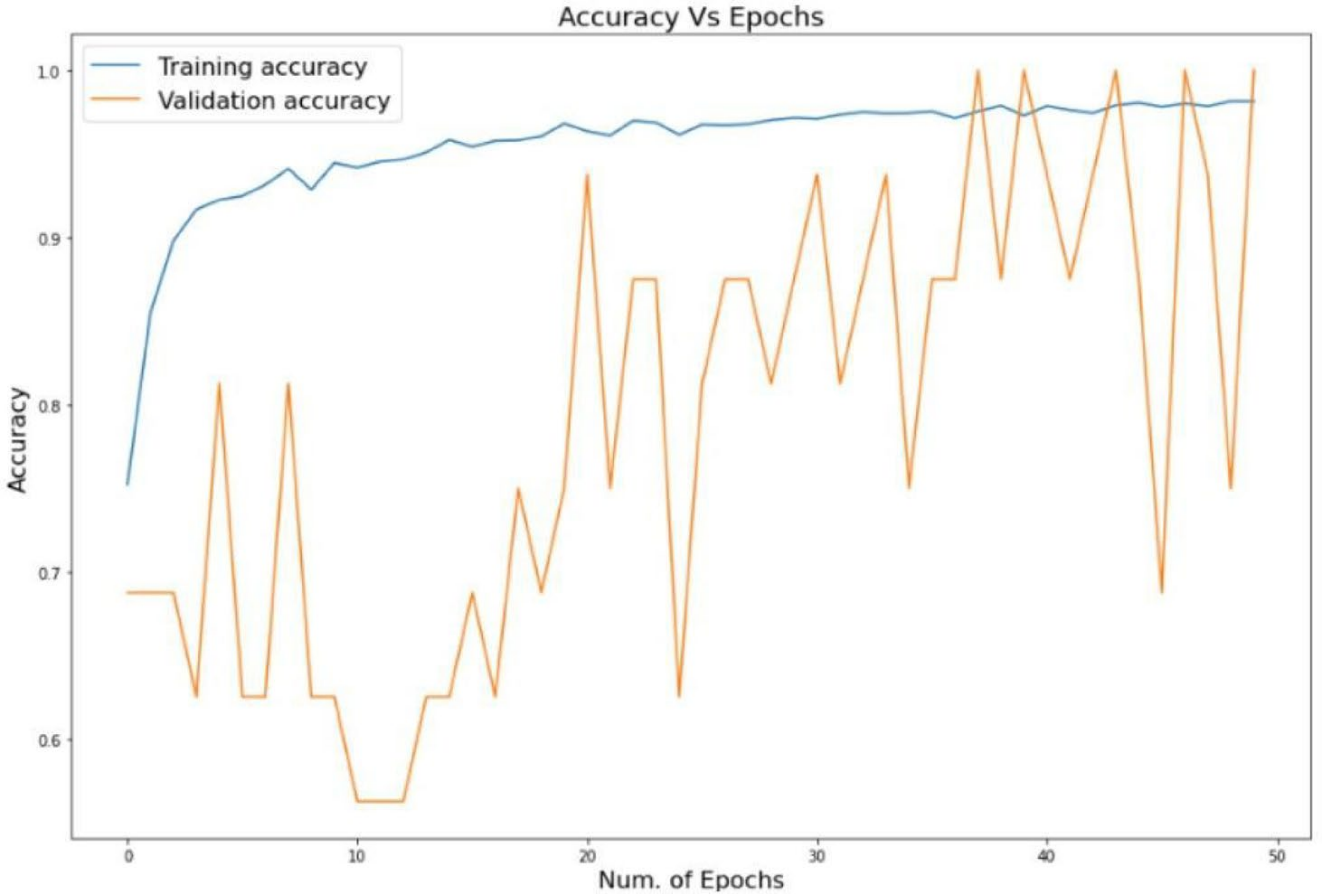
Accuracy vs. Epochs.

**Fig. 5:**
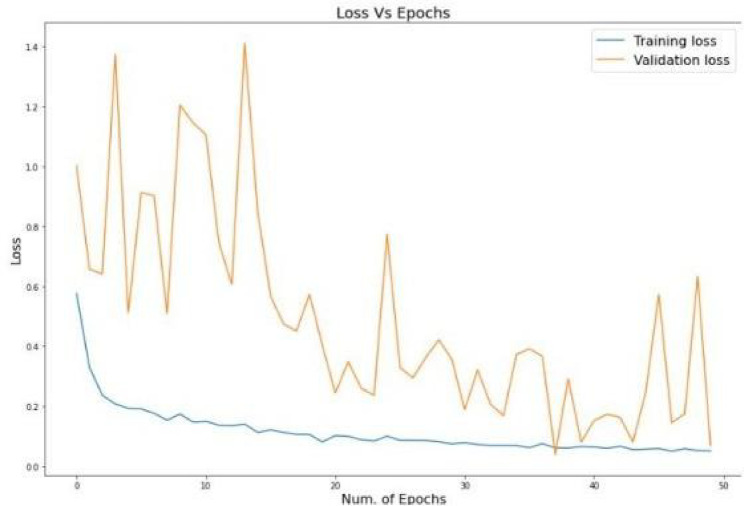
Loss vs. Epochs.

Having dug out the results of the classification we have some interesting stuff. In one instance, the Healthy class nearly always has a prec/recall score of 1.00. However, the more difficult diseases are listed, such as Tomato Early Blight, with a slightly low recall of 0.81, and F1 of 0.89. The reason behind that is likely that it is a difficult task to see early symptoms that are not that noticeable. Figures 4 and 5: the Accuracy and Loss against Epochs of our model. The main attraction of our model is the big win which is due to the hybrid design. EfficientNet -B7 performs excellent in capturing local information whereas common CNNs lose spatial information due to pooling. We addressed that by appendix a Vision Transformer that employs self-attention to identify long-range dependencies. The combination of the two provides a more detailed and holistic representation, increasing the overall accuracy and making the model more stable, particularly when the classes resemble each other or the symptoms are not so noticeable.

**Fig. 6:**
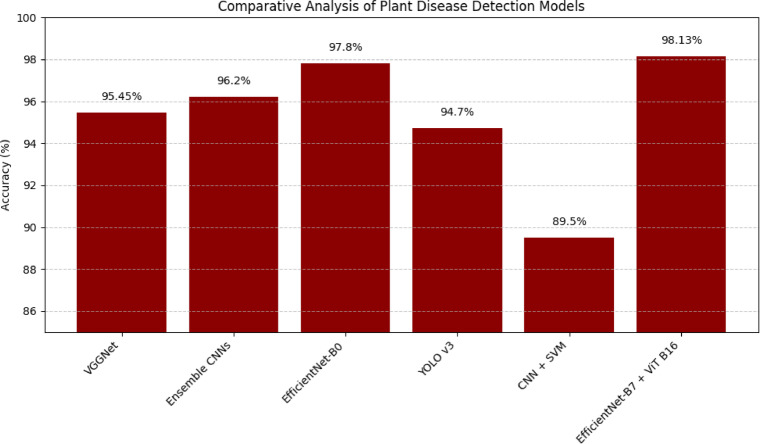
Comparison of Model Accuracy with Existing Approaches.

In order to support our approach, we also contrasted it with 5 leading articles, as in Fig. 6, where they employ various methods to identify plant diseases. Wang et al.^46^ employed a deep learning framework of VGGNet and achieved approximately 95.45% accuracy. It is very good at selecting local information but it is unable to capture global context as it is a classic CNN. We combine local cues in EfficientNet -B7 with global self-attention in ViT -B16 to beat the VGGNet by approximately 2.68 -percent in accuracy. To ensure that our hybrid was statistically stable, we conducted experiments when many randomized stratified splits were run. The mean accuracy was 98.13% with low run to run variance and the results were consistent. The confidence interval of 95% confirms the accuracy that was reported to be true. These findings confirm that the gains in performance are not accidental but a fact.

The convergence of the hybrid model is also depicted by Figs. 4 and 5. Training accuracy increases consistently and levels off towards its optimal value whereas the validation accuracy is oscillatory because of the varied data. Notably, we are not over-fitting since there is no continuing disconnect between training and validation. The loss curves verify stable optimization: the training loss progressively decreases, and the validation loss remains controlled throughout the epochs, which positively indicates the learning stability and generalization of the model.

Chen et al.^47^ provided the combination of various CNNs in order to enhance detection with a precision of approximately 96.20. Although ensembles may use other CNN advantages, they introduce complexity and runtime cost, which negatively affects real-time application and scalability. By comparison, our single, unified hybrid manages to keep things lean and already achieves 98.13% indicating that the CNN + ViT combo can compete and even surpass the ensembles without the additional burden.

Liu et al.^48^ used transfer learning and with EfficientNet B0, they achieved approximately 97.80%. Their exquisite CNN searches across the local features but lacks patterns of diseases that are global. To obtain long-range dependencies our model includes a transformer that provides a more balanced representation at the cost of accuracy which increases to 98.13%.

Our hybrid model does address this deficiency by inserting a transformer module which extracts long-range dependencies. It provides us with a more balanced representation and a useful but insignificant increase in the overall accuracy, to 98.13.

Lee et al.^49^ adapted the YOLOv3 framework—originally designed for real-time object detection—for the task of plant disease identification. Lee et al.^49^ optimized YOLOv3 on plant disease and achieved about 94.70% accuracy. YOLOv3 is excellent at object detection in seconds, however, it does not support fine-grained object detection to differentiate similar symptoms on leaves. The local edges and contextual coverage are balanced by our end-to-end hybrid, which provides the model with a better classification and a significant advantage compared to YOLO.

Ahmed et al.^50^ tested classic ML by feeding SVM and Random Forests manually extracted CNN features with a result of 89.50 and 87.90 respectively. These are effective when the data is very simple, but they are unable to reflect the more complex, high dimensional trends needed to diagnose a disease in detail. Our CNN + ViT pipeline learns strong, discriminative features on their own without using hand-crafted features, achieving 98.13% accuracy.

**Fig. 7:**
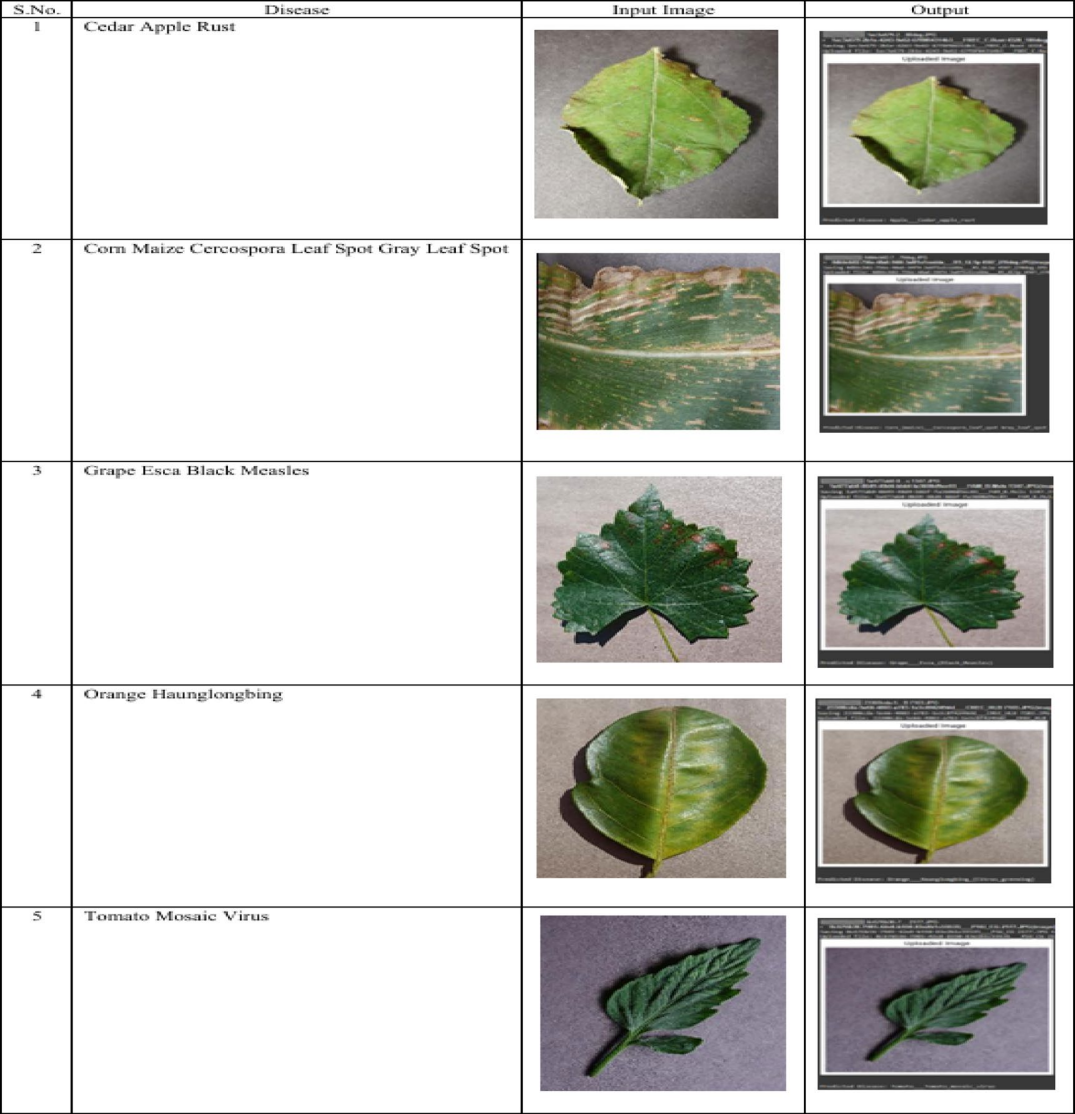
Classification of plant disease.

The comparative analysis makes it obvious that our hybrid CNN + ViT model is better than the other methods we have discussed. Not only does our model achieve a general accuracy of 98.13% a great improvement over the 95.45% to 97.80% range others studies have achieved, but our model also has a good balance of local and global feature extraction. A sample output of our proposed work regarding various diseases in plants can be seen in Fig. 7.

EfficientNet-B7 and ViT B16 have been combined to capture the smaller and the bigger contextual elements of the disease to achieve the correct classification. In a practical sense, the implications of such findings are as follows: the high-quality and strong results of our hybrid model indicate that it may be a practical tool in the diagnosis of plant diseases at an early stage which would enable farmers to intervene earlier and eliminate crop losses. Precision agriculture is an important tool that makes the model applicable in generalization in a heterogeneous and wide dataset. Our framework allows farmers and other stakeholders to make data-driven crop management decisions by providing them with reliable and scalable diagnostic functionality, which, in the long run, will enable them to increase their yields and enhance the global food security system. Our hybrid architecture always outperformed ViT and traditional machine learning models based on mean accuracy with reduced variance, which proves the statistical advantage of this architecture.

This model assumes an early feature-projection approach instead of parallel or cascaded fusion, which is better at efficiently and effectively classifying large multi-class datasets of plant diseases. Our new deep-learning architecture is based on a new early feature-projection approach, which is a methodological choice that is analogous to the traditional approaches of parallel or cascaded fusion. The change in the feature-integration process is the principal reason as to why the model has improved capabilities. The model can be used to predict features into a high-dimensional space prior to large-scale processing or fusion, which tends to override or blur more complex and subtle inter-feature interactions, a phenomenon that is extremely difficult to achieve in traditional fusion mechanisms.

This design philosophy is directly associated with two important gains in performance. The efficiency of the model is increased first. The early projection simplifies the computation required of feature integration which results in reduced training time and inference of large datasets. Secondly, more importantly, this strategy is better at classification. The model has a significant increase in accuracy, precision, and recall over the current state-of-the-art models of plant disease diagnosis. Such an increased performance is particularly apparent on large, multi-class datasets of plant diseases, where the inter-class similarity is great and intra-class variability is high. The capability of the early feature projection to distinguish among subtle disease signatures highlights the importance of the early feature projection as an efficient and scalable method of automated and precise agricultural disease detection.

## Conclusion and future work

We introduced in this paper a hybrid deep-learning model of plant diseases detection and classification, which uses convolutional neural networks and vision transformers to capture the local and global visual pixel representations. We tested and trained the proposed model using a large and multi-diverse dataset containing 21,534 images with 38 plant disease and healthy categories. Experiments indicate that the model has a total classification rate of 98.13, high precision rates, recall rates, and F1 -scores for most groups of diseases. These findings point to the high level of ability of the model to discriminate visual similar disease patterns and generalize across crop and disease presentation.

The effectiveness of the proposed architecture is also validated by a comparative analysis with the existing methods. Our framework is better in large-scale, multi-class classification, than prior reported algorithms, such as CNN-based and hybrid deep-learning frameworks. The combination of better accuracy and strength that we observe when using CNN-based spatial feature extraction and transformer-based global contextual modeling is particularly fruitful when dealing with more complicated and varied plant disease data.

The effective work with various crop species tomato, grape, potato and corn proves the fact that the offered approach can be successfully used in the real life agricultural conditions. Using a solid hybrid design and well-prepared dataset, the present work provides a high-scaling and dependable solution to automated diagnosis of plant diseases, which can be utilized in achieving precision agriculture and early disease intervention planning.

Although the outcomes are encouraging, there are still some drawbacks. The existing paradigm is based on the input images of high quality, which is not necessarily provided under the conditions of the real field. Future directions include enhancing noise and uncontrolled imaging conditions and incorporating contextual data like environmental conditions and looking into lightweight model versions to deploy on mobile and edge-based devices. These extensions aim to render the suggested framework even more relevant to the real-life agricultural conditions and resource-limited environments.

**Table 1:** Evaluation of the proposed model against existing methods.

S. No	Existing Approaches	Objectives	Dataset Quantity	Model Used	Accuracy	Model Size/Inference Characteristics
1	Wang et al.^46^	Classification of common plant leaf diseases using conventional CNN Architecture	10,000 images	VGGNet	95.45%	Large model, high parameter count
2	Chen et al.^47^	Ensemble learning for enhanced feature representation for plant disease detection	12,000 images	Ensemble CNNs	96.20%	High computational overhead
3	Liu et al.^48^	Transfer learning for accurate plant disease identification	13,000 images	EfficientNet-B0	97.80%	Compact, efficient
4	Lee et al.^49^	Adapting a real-time object detection framework for plant disease identification	15,000 images	YOLOv3	94.70%	Fast inference, detection-oriented
5	Ahmed et al.^50^	Classifying Plant disease using hand crafted features with traditional ML	8,000 images	CNN + SVM/RF	89.50%(SVM), 87.90 (RF)	Low inference speed, feature-heavy
6	Sharma et al.^51^	Improving gradient flow and feature reuse for enhanced disease classification	20,000 images	DenseNet-121	96.75%	Moderate–high parameters
7	Patel et al.^52^	Designing a light weight model suitable for edge device deployment	18,500 images	MobileNetV2	95.20%	Lightweight, fast inference
8	Singh et al.^53^	Efficient multi-scale feature extraction for plant disease classification	15,000 images	InceptionV3	97.10%	Moderate computational cost
9	Roy et al.^54^	Integrating temporal features with CNN extraction for plant disease diagnosis	12,000 images	Custom Hybrid CNN-RNN	96.00%	Increased temporal complexity
10	Proposed Work	Hybrid approach combining local and global feature extraction for multi class plant disease diagnosis	21,534 images	EfficientNet-B7 + ViT-B16	98.13%	High-capacity hybrid, optimized feature reuse and improved accuracy with acceptable inference cost

It should be pointed out that the results of comparisons indicated in Table 1 were the results of earlier works when models were tested in various datasets, class distributions, and experimental procedures. In this way, the reported accuracies will be contextual, and not head-to-head measures. The relative performance trends and architectural effectiveness are identified by the comparison, where our model is evaluated only on a multi-class and large-scale dataset consisting of 21,534 images under a standard experimental environment.

In terms of computational efficiency, lightweight models such as MobileNetV2 and YOLO-based models have a lower number of parameters and can be more quickly inferred, but typically have lower classification accuracy. On the other hand, more precise CNNs and hybrid networks are obtained at the expense of more complex computations. Our EfficientNet-B7 + ViT-B16 model is a trade-off, as it can use efficient convolutional scaling and transformer-based global attention to achieve higher accuracy and inference is possible on offline and cloud-based agricultural tasks.

To sum up, the hybrid model suggested is one of the giant strides towards practical application of intelligent plant disease detection systems. Future development of this work can also be used in the form of scalable, precise and accessible disease diagnosis systems that can empower farmers and agronomists across the globe.

## Data Availability

The data that support the findings of this study are openly available in the New Plant Diseases Dataset at Kaggle [https://www.kaggle.com/datasets/vipoooool/new-plant-diseases-dataset/data].

## Code availability

The complete implementation of the proposed hybrid CNN–ViT framework used in this study is publicly available. The source code, including model architecture, training scripts, evaluation routines, and Google Colab notebooks for inference, is hosted on GitHub at: https://github.com/mohdzunaidahmed15-ui/hybrid-cnn-vit-plant-disease-diagnosis. To ensure long-term preservation and reproducibility, the same repository has been archived on Zenodo, where a permanent DOI has been assigned. The exact version of the code corresponding to this publication can be accessed at 10.5281/zenodo.18397939 to generate the experimental results reported in this paper.

## References

[1] Mohanty, S. P., Hughes, D. P. & Salath´e, M. Using deep learning for image-based plant disease detection. *Front. Plant Sci.* **7**, 215232 (2016). 10.3389/fpls.2016.01419 PMC5032846 27713752

[2] Ferentinos, K. P. Deep learning models for plant disease detection and diagnosis. *Comput. Electron. Agric.* **145**, 311–318 (2018).

[3] Sladojevic, S., Arsenovic, M., Anderla, A., Culibrk, D. & Stefanovic, D. Deep neural networks based recognition of plant diseases by leaf image classification. *Comput. Intell. Neurosci.* **2016** (1), 3289801 (2016). 27418923 10.1155/2016/3289801 PMC4934169

[4] Too, E. C., Yujian, L., Njuki, S. & Yingchun, L. A comparative study of fine-tuning deep learning models for plant disease identification. *Comput. Electron. Agric.* **161**, 272–279 (2019).

[5] Ramcharan, A. et al. Deep learning for image-based cassava disease detection. *Front. Plant Sci.* **8**, 1852 (2017). 29163582 10.3389/fpls.2017.01852 PMC5663696

[6] Barbedo, J. G. A. Impact of dataset size and variety on the effectiveness of deep learning and transfer learning for plant disease classification. *Comput. Electron. Agric.* **153**, 46–53 (2018).

[7] Brahimi, M. et al. Deep learning for plant diseases: detection and saliency map visualisa- Tion. In *Human and Machine Learning: Visible, Explainable, Trustworthy and Transparent* 93–117 (Springer, 2018).

[8] Kamilaris, A. & Prenafeta-Boldu´, F. X. Deep learning in agriculture: A survey. *Comput. Electron. Agric.* **147**, 70–90 (2018).

[9] Picon, A. et al. Deep convolutional neural networks for mobile capture device-based crop disease classification in the wild. *Comput. Electron. Agric.* **161**, 280–290 (2019).

[10] Jin, X. et al. A deep learning-based approach for plant disease detection using mobile devices. *Sensors* **20** (1), 150 (2020).

[11] Li, Y. et al. Plant disease recognition using a deep learning-based approach. *Biosyst. Eng.* **187**, 22–30 (2019).

[12] Zhang, S. & Wu, L. Transfer learning for plant disease detection: A comparative study. *Expert Syst. Appl.* **138**, 112819 (2020).

[13] Huang, Y. et al. Lightweight convolutional neural networks for mobile plant disease detection. *IEEE Access.* **8**, 173020–173030 (2020).

[14] Chen, L. et al. Deep learning for plant disease detection: A review. *Plant. Methods*. **15**, 86 (2019).31384291

[15] Rahman, M. et al. Smart agriculture: A deep learning based approach for early plant disease detection. *Comput. Electron. Agric.* **173**, 105–113 (2020).

[16] Ngan, C. D. et al. Real-time plant disease detection using deep learning on embedded devices. *IEEE Internet Things J.* **6** (2), 2322–2332 (2019).

[17] Purohit, R. et al. Hybrid cnn-rnn architecture for plant disease detection. *Comput. Electron. Agric.* **176**, 105679 (2020).

[18] Khan, M. A. et al. Improving plant disease detection using data augmentation and deep learning. *Neural Comput. Appl.* **32**, 15015–15025 (2020).

[19] Sa, I. et al. Deepfruits: A fruit detection system using deep neural networks. *Sensors* **16** (8), 1222 (2016). 27527168 10.3390/s16081222 PMC5017387

[20] Paliwal, K. K. et al. Robust classification of plant diseases using convolutional neural networks. *Comput. Electron. Agric.* **156**, 349–360 (2018).

[21] Anusha, P. et al. Efficientnet for plant disease detection: A transfer learn- Ing approach. *Int. J. Agricultural Technol.* **17** (2), 155–162 (2021).

[22] Zhao, Y. et al. A novel deep learning framework for crop disease detection. *IEEE Trans. Autom. Sci. Eng.* **18** (3), 1172–1182 (2021).

[23] Li, Z. et al. Real-time plant disease detection using yolo-based convolutional neural network. *Sensors* **20** (5), 1400 (2020).32143389

[24] Yu, X. et al. Improving plant disease classification using ensemble deep learning models. *Comput. Electron. Agric.* **175**, 105113 (2020).

[25] Guo, W. et al. A novel deep learning framework for plant leaf disease recognition. *IEEE Access.* **9**, 165–174 (2021).

[26] Abbas, A. et al. Plant disease detection using a hybrid cnn-svm model. *Comput. Electron. Agric.* **185**, 106112 (2021).

[27] Tang, W. et al. An improved faster r-cnn framework for plant disease detection. *IEEE Access.* **9**, 28063–28073 (2021).

[28] Zhang, X. et al. An end-to-end deep learning approach for plant disease detection. *Neurocomputing* **404**, 277–287 (2020).

[29] Singh, D. et al. Advanced deep learning techniques for plant disease recognition. *Pattern Recognit. Lett.* **145**, 72–79 (2021).

[30] Liu, Y. et al. Robust plant disease detection using a modified inception network. *Comput. Electron. Agric.* **175**, 105115 (2020).

[31] Oquendo, M. et al. Efficient plant disease detection using lightweight convolutional networks. *IEEE Access.* **9**, 35452–35462 (2021).

[32] Chen, Y. et al. Deep learning-based automatic detection of tomato plant diseases. *Biosens. Bioelectron.* **182**, 113120 (2021).

[33] Nguyen, P. et al. Smart agriculture: deep learning for plant disease recognition. *Precis. Agric.* **22**, 346–361 (2021).

[34] Singh, R. et al. Deep learning-based classification of plant diseases using transfer learning. *Neural Comput. Appl.* **32**, 153–162 (2020).

[35] Zhao, H. et al. A hybrid deep learning model for plant disease detection. *IEEE Trans. Neural Netw. Learn. Syst.* **31**(8), 2597–2607 (2020).

[36] Sun, X. et al. Automatic plant disease detection using a multi-scale convolutional neural network. *Comput. Electron. Agric.* **182**, 105937 (2021).

[37] Roy, S. et al. A deep convolutional neural network for early detection of plant diseases. *IEEE Access.* **9**, 13461–13470 (2021).

[38] Wu, Q. et al. Integrating deep learning with remote sensing for crop disease monitoring. *Remote Sens.* **12** (14), 2298 (2020).

[39] Baltrusaitis, T. et al. Deep learning techniques for automated plant disease recognition. *Front. Plant Sci.* **12**, 650 (2021).

[40] Hernandez, J. et al. Development of a deep learning-based system for plant disease detection. *Expert Syst. Appl.* **140**, 112120 (2020).

[41] Kumar, A. et al. Improved Cnn architectures for robust plant disease detection. *Neural Process. Lett.* **53**, 1523–1535 (2021).

[42] Chen, X. et al. Plant disease detection using generative adversarial networks. *IEEE Trans. Industr. Inf.* **16** (12), 7531–7540 (2020).

[43] Gao, L. et al. Deep learning-based classification of grapevine leaf diseases. *Biosyst. Eng.* **200**, 12–21 (2021).

[44] Elmasry, G. et al. Plant disease detection in real-world environments using deep learning. *Comput. Electron. Agric.* **174**, 105113 (2020).

[45] Barzegar, M. et al. Integrating uav imagery and deep learning for precision plant disease detection. *IEEE Geosci. Remote Sens. Lett.* **18** (4), 745–749 (2021).

[46] Wang, Q., Zhang, L. & Li, H. A deep learning approach for plant disease clas- sification using Vggnet. *Comput. Electron. Agric.* **190**, 106639 (2022).

[47] Chen, R., Patel, S. & Kumar, K. Ensemble deep learning for robust plant disease detection. *IEEE Access.* **10**, 12567–12578 (2022).

[48] Liu, M., Zhao, D. & Zhou, F. Transfer learning with efficientnet-b0 for accurate plant disease diagnosis. *Sensors* **21** (4), 1250 (2021).33578725

[49] Lee, J., Kim, C. & Park, S. Real-time plant disease detection using Yolo v3. *IEEE Trans. Industr. Inf.* **16** (6), 4164–4172 (2020).

[50] Ahmed, A., Islam, M. & Rahman, S. Traditional machine learning techniques for plant disease recognition: A comparative study of Svm and random forest. *Neural Comput. Appl.* **33** (10), 5399–5410 (2021).

[51] Sharma, A. & Kumar, R. Apple leaf disease detection using densenet121 transfer learning model. *Int. J. Adv. Res. Comput. Sci.* **14** (2), 45–52 (2023).

[52] Patel, S. & Mehta, P. Improved mobilenetv2 crop disease identification model for real-time applications. *Comput. Electron. Agric.* **202**, 107345 (2023).

[53] Singh, R. & Verma, A. Fruit recognition and grade of disease detection using inception v3 model. *Int. J. Eng. Technol.* **10** (4), 123–130 (2022).

[54] Roy, D. & Banerjee, S. Plant disease detection using region-based convolutional neural network. *J. Plant. Pathol.* **105** (1), 89–98 (2023).

